# Targeting Adenosine in Cancer Immunotherapy to Enhance T-Cell Function

**DOI:** 10.3389/fimmu.2019.00925

**Published:** 2019-06-06

**Authors:** Selena Vigano, Dimitrios Alatzoglou, Melita Irving, Christine Ménétrier-Caux, Christophe Caux, Pedro Romero, George Coukos

**Affiliations:** ^1^Department of Oncology, Ludwig Institute for Cancer Research Lausanne, Lausanne University Hospital and University of Lausanne, Lausanne, Switzerland; ^2^Department of Immunology Virology and Inflammation, INSERM 1052, CNRS 5286, Léon Bérard Cancer Center, Cancer Research Center of Lyon, University of Lyon, University Claude Bernard Lyon 1, Lyon, France; ^3^Department of Oncology, University of Lausanne, Lausanne, Switzerland

**Keywords:** adenosine, cAMP, CD73, CD39, cancer immunotherapy, T cells, tumor microenvironment

## Abstract

T cells play a critical role in cancer control, but a range of potent immunosuppressive mechanisms can be upregulated in the tumor microenvironment (TME) to abrogate their activity. While various immunotherapies (IMTs) aiming at re-invigorating the T-cell-mediated anti-tumor response, such as immune checkpoint blockade (ICB), and the adoptive cell transfer (ACT) of natural or gene-engineered *ex vivo* expanded tumor-specific T cells, have led to unprecedented clinical responses, only a small proportion of cancer patients benefit from these treatments. Important research efforts are thus underway to identify biomarkers of response, as well as to develop personalized combinatorial approaches that can target other inhibitory mechanisms at play in the TME. In recent years, adenosinergic signaling has emerged as a powerful immuno-metabolic checkpoint in tumors. Like several other barriers in the TME, such as the PD-1/PDL-1 axis, CTLA-4, and indoleamine 2,3-dioxygenase (IDO-1), adenosine plays important physiologic roles, but has been co-opted by tumors to promote their growth and impair immunity. Several agents counteracting the adenosine axis have been developed, and pre-clinical studies have demonstrated important anti-tumor activity, alone and in combination with other IMTs including ICB and ACT. Here we review the regulation of adenosine levels and mechanisms by which it promotes tumor growth and broadly suppresses protective immunity, with extra focus on the attenuation of T cell function. Finally, we present an overview of promising pre-clinical and clinical approaches being explored for blocking the adenosine axis for enhanced control of solid tumors.

## Introduction

IMT has led to unprecedented clinical success for some advanced cancer patients and has been accepted as a new pillar of cancer therapy (1). Thus, the identification of biomarkers predicting response to IMT, as well as the development of combinatorial strategies for increasing its effectiveness in more patients, and against a broader range of tumor-types, have become important areas of research (2). The nucleoside adenosine, involved in the regulation of multiple diverse physiological processes either as an intracellular metabolite of nucleic acid synthesis and energy-charge regulation or as an intercellular messenger in neurological, cardiovascular and immunological systems, has recently emerged as a major immuno-metabolomic checkpoint in tumors (3). Conditions of stress, such as hypoxia, lead to the accumulation of extracellular adenosine, predominantly derived from enzymatic ATP catabolism, which can act directly on tumor cells expressing adenosine receptors to promote their growth, survival and dissemination. In addition, adenosine, which under physiological conditions serves as an immuno-regulatory molecule to protect normal tissues from uncontrolled inflammation, can impair anti-tumor immunity, both through the attenuation of protective immune cells including T cells, NK cells, and dendritic cells (DCs), and by enhancing the suppressive capacity of T regulatory cells (Tregs), and myeloid-derived suppressor cells (MDSCs), amongst others. Here we review the targeting of the adenosine pathway to promote immune function and tumor control, with focus on T-cell activity, important experimental findings and an overview of clinical testing.

## Regulation of Adenosine Levels in Healthy vs. Malignant Tissue

Extracellular adenosine, a nucleoside and derivative of ATP, is involved in the regulation of diverse physiological processes including vasodilation (4), kidney-exerted water reabsorption (5), pain perception (6), and fine-tuning of the sleep–wake cycle (7). Even though levels of extracellular adenosine within healthy tissues are negligible (8–11), upon injury this nucleoside sharply accumulates at the interstitium where it potently restricts immune responses (12) and directly promotes wound healing (13). Under homeostatic conditions in healthy tissues, the cytosolic concentration of ATP ranges from 1 to 10 mM (14), while its extracellular levels are negligible (15). This sharp gradient can be rapidly disrupted however upon breaches of the plasma membrane induced by necrosis, apoptosis or mechanical stress, as well as by regulated ATP efflux. The latter, induced by a variety of stimuli including hypoxia, ischemia and inflammation, has been shown to extensively occur *via* exocytosis, transmembrane transfer through ATP-binding cassette (ABC) transporters, as well as by diffusion through a variety of anion channels or non-selective plasma membrane pores formed by connexins, pannexin-1 or the ATP receptor P2X7R (16–18). For instance, stimulated T cells release ATP through pannexin-1 hemi-channels and *via* exocytosis (19, 20).

**Figure 1:**
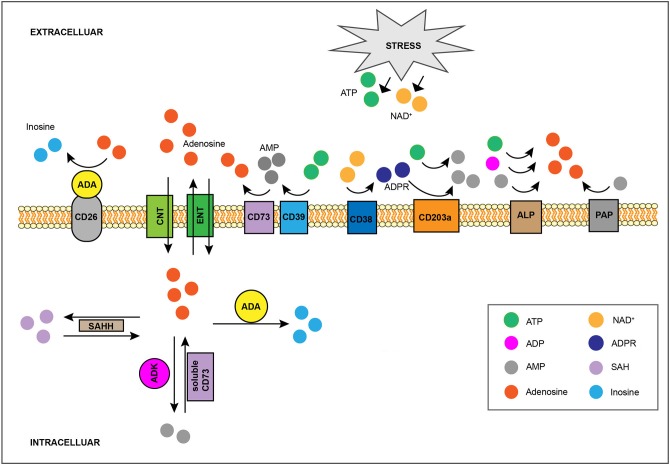
Regulation of interstitial adenosine levels in injured tissue. Stress-induced, extracellular buildup of ATP or NAD^+^ fuels catabolic adenosine-generating pathways, such as the one mediated by CD39 and CD73. The activity of other ecto-nucleotidases including CD38, CD203a, ALP, and PAP, also contribute toward extracellular adenosine accumulation. Adenosine can also be produced intracellularly by SAHH-exerted hydrolysis of SAH, as well as by soluble CD73-mediated catabolism of AMP, and it can be exported by ENTs in a diffusion-limited manner. On the flip side, the combination of CD26-bound ADA activity and of adenosine cellular uptake, either through equilibrative ENTs or via concentrative CNTs, limits interstitial adenosine levels. Intracellularly, adenosine can be eliminated via its conversion to SAH by SAHH, to AMP by ADK, or to inosine by ADA. SAHH, S-adenosylhomocysteine hydrolase; SAH, S-Adenosylhomocysteine; ENTs, equilibrative nucleoside transporters; CNTs, concentrative nucleoside transporters; ADK, adenosine kinase; ADA, adenosine deaminase.

Once in the extracellular space, ATP undergoes rapid stepwise dephosphorylation by ecto-nucleotidases (21, 22) including the E-NTPDase CD39, which converts ATP or ADP to ADP or AMP, respectively, and the 5′-nucleotidase CD73, which dephosphorylates AMP to adenosine (18, 23) (Figure 1). Additional enzymes whose ecto-activity contributes toward extracellular adenosine generation are other E-NTPDases, members of the ecto-phosphodiesterase/pyrophosphatase (E-NPP) family, nicotinamide adenine dinucleotide (NAD^+^) glycohydrolases, the prostatic acid phosphatase (PAP), and the alkaline phosphatase (ALP) (21) (Figure 1). Briefly, the co-enzyme NAD^+^, another key cellular component whose extracellular concentration significantly rises in injured tissue (24, 25), is converted to adenosine diphosphate ribose (ADPR) by the NAD^+^ glycohydrolase CD38 (26), while ADPR as well as ATP are metabolized to AMP by the E-NPP CD203a (27). Moreover, PAP, which is predominantly, but non-exclusively, expressed in prostate tissue (28), is capable of converting extracellular AMP to adenosine (29), whereas ALP catalyzes the hydrolysis of ATP, ADP and AMP to adenosine (21). Finally, adenosine can also be produced intracellularly either by S-adenosylhomocysteine hydrolase (SAHH)-exerted hydrolysis of S-Adenosylhomocysteine (SAH), a metabolite of the transmethylation pathway, or due to soluble CD73-mediated catabolism of AMP, a nucleoside participating in multiple cellular processes and whose concentration rises within cells of low energy charge (30) (Figure 1). Intracellularly-generated adenosine can be secreted in a diffusion limited-manner through bidirectional equilibrative nucleoside transporters (ENTs) (31). However, although there is evidence suggesting that hypoxia can boost intracellular adenosine production (32, 33), the contribution of this pathway toward injury-caused interstitial adenosine buildup is considered minor due to concurrent hypoxia-induced downregulation of the aforementioned transporters (34, 35). Given its diverse effects, adenosine presence at the extracellular space is subject to tight spatiotemporal control (12, 13, 36). For instance, extracellular accumulation of adenosine is counteracted by its inward transfer through ENTs or concentrative, sodium gradient-dependent, symporters (31) as well as by the function of intra/extracellular adenosine deaminase (ADA) and of cytosolic adenosine kinase (ADK), which respectively convert adenosine to inosine or AMP (37) (Figure 1).

In contrast to homeostatic conditions, ATP levels are highly elevated in the TME as a result of necrosis, apoptosis, hypoxia, and persistent inflammation (17, 18), and intra-tumoral adenosine levels can reach micromolar concentrations (9, 10, 38). ATP catabolism in tumors is primarily mediated by CD39 and CD73 (39–41), and high expression of these ecto-nucleotidases is strongly associated with poor clinical outcome for patients suffering a variety of cancer-types (3, 42, 43). In particular, CD39 and/or CD73 (over)expression has been detected on the surface of tumor cells (39, 44–51), cancer-associated fibroblasts (CAFs) (52–54), mesenchymal stem cells and stromal cells (55–57), endothelial cells (ECs) (45, 46, 51), myeloid derived suppressor cells (MDSCs) (58–60), tumor associated macrophages (TAMs) (53, 61), Tregs (46, 62–64), Th_17_ cells (65) and of antigen experienced/exhausted conventional CD4^+^ and CD8^+^ T cells (64, 66–68). In addition, CD39/CD73-bearing exosomes (69, 70), released by tumor cells (71), Tregs (72), and MDSCs (57, 73) further contribute to adenosine generation. Currently, hypoxia as well as incessant inflammation are considered to be the main drivers of intra-tumoral CD39 and CD73 overexpression. Namely, hypoxia-induced (74, 75) HIF1α (76–79) and Sp1 (80) activity promotes expression of these ecto-nucleotidases. Along the same lines, signaling pathways initiated by inflammation-associated molecules, such as IL-2 (81), IL-6 (66, 82), IL-1β (83), TNFα (83–85), type I IFNs (86, 87), IL-27 (66, 88), TGFβ (82, 89, 90) as well as by inducers of the Wnt (91, 92) or cAMP (83, 93–95) signaling pathways also boost CD39 (66, 81, 82, 88, 89, 95) and CD73 (81–87, 89–94) levels.

Although CD39 and CD73-mediated catabolism of extracellular ATP is considered to account for the bulk of intra-tumoral adenosine generation, expression levels of ecto-enzymes participating in alternative adenosine production pathways also rise in the advent of cancer. For instance, CD38 is frequently upregulated within neoplastic tissues (26, 96, 97) and sporadic evidence suggests that CD203a levels also increase on TME components (98, 99). Along the same lines, the serum concentration of PAP increases during prostate cancer progression (100) while others suggest it gets upregulated on cancerous tissue as well (28). Finally, several studies have demonstrated elevated levels of ALP on cancer cells (101, 102) as well as a correlation of serum ALP levels and disease stage (103–105). Critically, the relative contribution of these alternative adenosine-producing pathways toward intra-tumoral buildup of this nucleoside remains to be determined. Finally, along with aberrant production, defective uptake resulting from the down-modulation of equilibrative (106, 107) as well as concentrative (108–110) nucleoside transporters, also driven by hypoxia (34, 35, 111), further contributes to adenosine accumulation in the TME.

## Adenosine Receptor Signaling

Four adenosine receptors (ARs), all coupled to G-proteins, have been identified; A1R, A2AR, A2BR, and A3R (112, 113). While A1, A2A, and A3 are described as high affinity adenosine receptors (EC_50_ in the range of 0.1–0.7 μM), A2BR is considered as low affinity because it is activated only in the presence of high concentrations of adenosine (EC_50_ of 15–25 μM), such as may be found in the TME or under other pathological conditions. Upon adenosine binding, these GPCRs induce the replacement of GDP bound by the heterotrimeric G proteins, a class of GTP hydrolases, with GTP thus resulting in the dissociation of the latter into Gα monomers and Gβγ dimers, now free to modulate downstream effectors before their GTP hydrolysis-induced re-association (114).

Of the four classes of Gα proteins characterized to date, namely Gα_s_, Gα_i_, Gα_q/11_, Gα_12/13_, only Gα_s_ and Gα_i_ directly influence the activity of adenylyl cyclases (AC), enzymes that catalyze the cyclization of intracellular ATP into cyclic adenosine monophosphate (cAMP) (114). In terms of function, triggering of the Gα_s_-coupled A2AR and A2BR promotes AC activity (115). In contrast, stimulation of the Gα_i_-paired A1R and A3R inhibits cAMP generation (115). Although modulation of intracellular cAMP content constitutes a crucial aspect of extracellular adenosine-exerted regulation, stimulation of its receptors induces a variety of cAMP-independent biochemical effects, such as A1R/Gα_i_, A2BR/Gα_q/11_, A3R/Gα_q/11_-induced stimulation of phospholipase C (PLC) activity and A1R/Gα_i_, A2AR, A2BR/G_q/11_, A3R-mediated ERK activation (115). Finally, elevation of extracellular adenosine levels induces receptor-independent boosting of AMP-activated protein kinase (AMPK) via intracellular transfer of this nucleoside followed by its conversion to AMP (116, 117).

## Adenosine-Induced Intracellular cAMP Accumulation Impairs T Cell-Mediated Antitumor Responses

It is now understood that T cells play a major role in tumor control (118–120). As will be discussed however, elevated levels of adenosine in the TME can potently impair T-cell function by inducing accumulation of intracellular cAMP.

### Levels of Adenosine Receptors on the T Cell Surface

Murine (121–127) and human (128–132) T cells express all four ARs, and levels of A2AR (122, 124–127, 129), A2BR (126, 127, 130), and A3R (127, 131) increase upon T cell activation. However, the biology of T cells is primarily affected by the predominantly expressed A2AR (122, 123, 128, 132). Of note, similarly to CD39 and CD73, A2AR, and A2BR are upregulated due to hypoxia-induced HIF1α (133) transcriptional activity. Moreover, mRNA levels of both A2AR and A2BR are upregulated in T cells specifically upon provision of anergic stimulus (134). Validating these findings, adoptively transferred tumor-specific T cells isolated from tumors contained twice the A2AR mRNA levels than counterpart T cells isolated from spleens of tumor-bearing mice (135). Since triggering of the different ARs initiates diverse and even antagonistic signaling pathways, the net cellular effects of adenosine are determined by the relative surface expression of its receptors. It is clear, however, that treatment of human (136, 137) or murine (38, 126, 138, 139) T cells with adenosine or adenosine analogs induces A2AR- (38, 126, 137–139) as well as A2BR- (38, 136) mediated intracellular cAMP build-up.

### The Mechanics of cAMP-Mediated T Cell Suppression

The secondary messenger of adenosine cAMP, also a derivative of ATP, is involved in a diverse range of cellular functions including metabolism, transcription, and growth, while oscillations of its levels within distinct cell populations are paramount for the regulation of multiple bodily functions, such as endocrine, cardiovascular, neuronal, and immune processes (140). The intracellular concentration of cAMP is determined by the antagonistic activities of ACs, and of cAMP-specific phosphodiesterases (PDEs), proteins that hydrolyze cAMP to 5′-AMP. Although cAMP can diffuse within the cytosol, the co-localization of the highly-targeted AC and PDE activities in particular subcellular regions results in the formation of distinct cAMP microdomains within which co-localized cAMP effectors are activated by *in-situ* generated cAMP before its swift degradation (141, 142). The formation of such microdomains is mediated by AKAPs, scaffold proteins shown to bind ACs, PDEs as well as effectors of the cAMP-signaling pathway (143, 144). Of the 10 currently identified AC isoforms, T cells express AC3, AC6, AC7 and AC9 (145, 146) with most cAMP production catalyzed by AC7 (146). As previously described, A2AR and A2BR are coupled to Gα_s_ which stimulates the activity of ACs. Of the 11 PDE families characterized to date, isoforms belonging to the relatively strong-affinity (147) cAMP-binding families of PDE1 (145, 148), PDE3 (145, 149), PDE4 (145, 149), PDE7 (145, 149–151), PDE8 (145, 151, 152), and PDE11 (145) have been observed within T cells, with most cAMP hydrolysis carried out by PDE3 and PDE4 isoforms (148, 149, 153). Of note, cAMP levels in T cells can also be augmented by additional factors in the TME including prostaglandin E_2_ (PGE_2_) (154), norepinephrine (155), histamine (156), the neuropeptides VIP and PACAP (157, 158), and low pH (159). Additional phenomena contributing toward cAMP build-up within effector T cells include TCR triggering (160, 161) as well as direct cAMP transfer by tumor cells (162) or Tregs (163) *via* gap junctions.

Accumulation of cAMP within the T cell cytosol induces the activity of protein kinase A (PKA) and of exchange protein directly activated by cAMP (EPAC). PKA, the dominant effector of the cAMP signaling pathway (164) is an heterotetramer comprising two catalytic (C) subunits, maintained in an inactive state by tethering to two regulatory (R) subunits (165). Binding of cAMP to the R-subunits induces a conformational change resulting in the release of the C-subunits (166). As a result, liberated PKA C-subunits within T cells phosphorylate a wide variety of substrates affecting multiple signaling pathways (167). It is well established that sustained PKA activity disrupts signaling induced by triggering of the TCR, of the co-stimulatory receptor CD28 (168, 169) as well as by the IL-2 receptor (IL-2R) (170). Negative regulators of these signaling pathways, whose activity is bolstered by PKA, include Csk (171), SHP-1 (172), SHIP1 (173), HPK1 (174), and PP2A (175). Conversely, PLCγ1 (176, 177), Raf-1 (178, 179), JAK3 (170), RhoA (180, 181), VASP (182) as well as the transcription factors NFAT (183, 184) and NFkB (185, 186) constitute mediators or endpoint effectors of the aforementioned axes whose activity is dampened by PKA.

PKA activity also significantly affects cytoplasmic potassium concentration within T cells by inhibiting the activity of Kv1.3 (187) and KCa3.1 (188, 189), channels which are responsible for the bulk of potassium efflux by T cells (190). In a negative-feedback fashion, PKA induces reduction of the cytosolic cAMP concentration by directly phosphorylating AC6 in an inhibitory fashion (191) as well as isoforms of PDE3 (192), PDE4 (193, 194), PDE8A (195) in a stimulatory manner. At the transcriptional level, PKA augments the activity of CREB cAMP responsive element binding (CREB), cAMP responsive element modulator (CREM) and activating transcription factor-1 (ATF-1) (196), which induce or counteract the transcription of multiple inflammation-relevant genes such as IL-2 (197–199), IFNγ (200–202), IL-4 and IL-13 (203, 204), IL-17 (205–208), and FoxP3 (209, 210). Specifically, PKA promotes the transcriptional activity of CREB by phosphorylating it thus increasing its affinity for its co-activators CBP and p300 (211), and by promoting the nuclear localization of CRTC (212), another family of CREB co-activators. Finally, PKA directly phosphorylates and activates ATF-1 (213) as well as distinct CREM isoforms (214) in a way similar to CREB.

The guanine nucleotide exchange factor EPAC1 is another effector of cAMP in T cells (215, 216). cAMP binds to the cAMP-responsive N-terminal region of EPAC1 and induces an open conformation rendering its catalytic core accessible to its effectors (217, 218). The most heavily characterized EPAC1 effector in T cells is the anergy-associated GTPase Rap1 (219, 220) which in its GTP-bound form is targeted to the plasma membrane (221) where it inhibits TCR-induced MEK-ERK activation by sequestering Raf-1 (220, 222).

### Overview of the Inhibitory Effects of cAMP on T-Cell Biology

A variety of molecules, including cAMP analogs, direct AC activators (e.g., forskolin and cholera toxin) and PDE inhibitors have been used to elucidate the diverse effects of intracellular cAMP accumulation on T-cell biology. In the presence of such molecules (223–228) as well as by A2AR triggering (125, 126, 229) the capacity of previously unstimulated T cells, CD4^+^ or unfractionated, to differentiate post-activation toward cells that produce Th1 (125, 126, 223–225, 229) or Th2 (226–229)-signature cytokines is drastically diminished. This occurs in a PKA-dependent fashion (230, 231) through multi-level disruption of TCR- or CD28-induced signaling (122, 232). Intriguingly, A2AR agonist-induced impairment of IFNγ production remains evident even when A2AR agonist-pretreated T cells are re-stimulated in the absence of this agent (139). Furthermore, agents that directly activate the cAMP pathway (233–235), as well as adenosine (122, 138, 232, 236, 237), have been shown to restrict stimulation-induced AKT activation (122, 232, 233, 238) and to induce stabilization of β-catenin, which restricts maturation toward terminally differentiated effector cells (239). Moreover, such agents can prevent FasL upregulation, thus averting FasL-mediated activation-induced cell death (AICD) (127, 138, 235, 237). Finally, such molecules abolish mitogenic-stimulus-induced T cell proliferation, in a PKA-dependent manner (240), by downmodulating the transmission of TCR/ CD28- and IL-2 (241)-initiated signaling, as well as IL-2 production (126, 229, 231) and IL-2Ra expression (242).

Forskolin, cAMP analogs, PDE inhibitors (152, 243–245) and adenosine (188, 246–248) also diminish T cell adherence (152, 243, 246, 248) by down-modulating the expression levels of ICAM-1 (249, 250) as well as of the integrins α_4_ (251, 252) and β_2_ (251, 253), components of VLA-4 and LFA-1, respectively. Such agents also impair T-cell migration (188, 244, 245, 247) by inducing KCa3.1 inhibition (188, 189). In addition, cAMP-mediated signaling (230, 254, 255) or the presence of A2AR agonists (139, 168, 230, 231) diminishes T cell cytotoxicity, in a PKA-dependent manner (168, 230, 231), probably as a result of impaired TCR signaling, motility/adhesion, granule exocytosis (138), as well as due to decreased expression of FasL, Granzyme B (GzB), and perforin (127).

Lastly, cholera toxin (256), PDE inhibitors (257–259), forskolin (157) and A2AR agonists (126, 260) not only skew T cells toward the Treg lineage *via* induction of FoxP3 expression (126, 256–258, 260), but also enhance the capacity of Treg cells to suppress responder T cells (258–260), at least in part by upregulating CTLA-4 levels (157, 260). Thus, cAMP can potently diminish the differentiation and effector activities of CD4^+^ and CD8^+^ T cells, while promoting the differentiation toward Tregs, as well as their suppressive capacity.

## The Pleiotropic Effects of Adenosine in the Tumor Microenvironment

**Figure 2:**
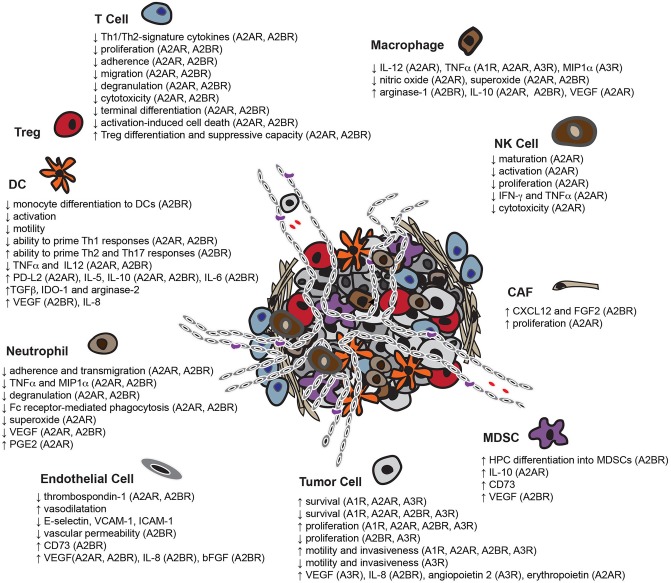
Overview of the pleiotropic effects of adenosine in the TME. Adenosine enables tumor cells to escape immune-surveillance by limiting the functionality of multiple potentially protective immune infiltrates including T cells, DCs, NK cells, macrophages and neutrophils, while enhancing the activity of immunosuppressive cell-types, such as MDSCs and Tregs. In addition, adenosine not only assists tumor cells in co-opting adjacent fibroblasts for support, but also induces the formation of new blood vessels. Adenosine also affects the capacity of some tumor cell-types to survive, proliferate, migrate and invade neighboring tissues (HPC, bone marrow-derived hematopoietic progenitor cells).

Along with T cells, many other cell types in the TME including other protective or suppressive immune infiltrates, tumor-associated fibroblasts, endothelial cells and cancer cells also express functional ARs (3, 261–266). Here we briefly describe the effects of adenosine-induced signaling on them (Figure 2).

### Dendritic Cells

The biology of DCs, specialized antigen presenting cells (APCs) and critical messengers between the innate and adaptive immune system, can be severely impaired by adenosinergic signaling. For example, it has been reported that adenosine binding to A2BR (267) halts the differentiation of monocytes to DCs (267, 268). In addition, adenosine averts inflammatory stimulus-induced DC activation (269), whereas A2AR (270) and A2BR triggering (267, 271, 272) diminishes the capacity of DCs to prime Th1 immune responses (267, 270, 271) but rather prompts DCs to skew naïve T cell differentiation toward Th2 (267, 271) and Th17 (272) lineages. Adenosine-treated DCs exhibit decreased expression or secretion of TNFα and IL-12 (268–271, 273) and enhanced production of IL-5 (270), IL-10 (267, 268, 270, 273), IL-6 (267, 272) and TGFβ (267). Moreover, such DCs are less motile due to chemokine receptor downregulation (274), and have a tolerogenic effect on the TME due to overexpression of TGFβ (267), IL-10, IDO-1 (267), arginase-2 (267, 275), as well as A2AR-mediated upregulation of PD-L2 (276). Finally, adenosine compels DCs to secrete the proangiogenic factors VEGF (267, 275) in an A2BR-dependent manner as well as IL-8 (267).

### Macrophages

Stimulation of adenosine receptors hinders the differentiation of monocytes to macrophages, probably through cAMP accumulation (277). Moreover, by engaging A1R (278), A2AR (278–282), A3R (281, 283) or setting off Gα_s_-paired ARs (284), adenosine reduces the pro-inflammatory activity of macrophages by dampening their ability to produce IL-12 (279), TNFα (278–280, 282, 283), macrophage inflammatory protein-1α (MIP1α) (281), nitric oxide (278, 285) and superoxide (284). In addition, by triggering A2AR (282, 286, 287), A2BR (288, 289) or unidentified ARs, adenosine promotes an M2 polarization of macrophages by inducing upregulation of arginase-1 (288, 290), IL-10 (279, 286, 289) and VEGF production (282, 287).

### NK Cells

A2AR stimulation by adenosine not only restricts the NK maturation (291), but also their capacity for stimulus-induced CD69 upregulation (292, 293), proliferation (291, 294) as well as IFNγ (292, 293) and TNFα (294, 295) production. Furthermore, largely *via* A2AR triggering, adenosine diminishes target cell killing by NK cells (292, 294, 296–298).

### Neutrophils

Adenosine exerts a variety of inhibitory effects on neutrophils. For example, triggering of A2AR (299–303), A3R (304), non-specified A2Rs (304–307) or ARs dampens their ability to adhere (299, 305, 308, 309), transmigrate (310), secrete TNFα and MIP1α (300, 306), degranulate (301, 302, 304, 311), perform Fc receptor-mediated phagocytosis (307) and produce superoxide (299, 301–303). Interestingly, others claim that A2AR and A2BR signaling has been shown to suppress VEGF production (310). Finally, A2AR stimulation prompts neutrophils to secrete higher levels of PGE2 (312).

### MDSCs

A2BR-mediated signaling boosts differentiation of bone marrow hematopoietic progenitors toward a tolerogenic myeloid-derived cell subset, the MDSCs (313). Moreover, A2AR activation promotes IL-10 production by MDSCs (314) and treatment with an adenosine analog results in increased expression of CD73 (313). Finally, it has also been shown that A2BR stimulation on MDSCs augments VEGF production (315).

### Stromal Cells

Adenosine, along with critically contributing to the establishment of a tolerogenic TME, also enables tumors to subvert fibroblasts into supporting them and to induce formation of new blood vessels, processes essential to their growth and dissemination. CAFs, for example, are stromal cells that support tumors by secreting the pro-metastatic and angiogenic (316) chemokine CXCL12 (317), as well as the mitogenic (318) fibroblast growth factor 2 (FGF2) (319). Triggering of A2BR on the surface of CAFs boosts expression of both CXCL12 and FGF2 (320) whereas A2AR-induced signaling stimulates their proliferation (54). As previously mentioned, adenosine can stimulate VEGF secretion by multiple cell types found within the TME, which in turn promotes angiogenesis by supporting the survival, migration and proliferation of endothelial cells (321, 322). It has also been shown that A2AR (323) and A2BR (66) stimulation diminishes production of the anti-angiogenic factor thrombospondin-1 by endothelial cells. Furthermore, adenosine not only augments the rate of intra-tumoral nutrient delivery by inducing vasodilatation (324), but also hinders leukocyte extravasation (325) through downregulation of adhesion molecules, such as E-selectin (326, 327) VCAM-1 (326, 327) and ICAM-1 (327, 328) on the surface of endothelial cells, as well as by limiting vascular permeability (325, 327, 329–331) through A2BR activation (329–331). Finally, signaling initiated by triggering of A2AR (332, 333), A2BR (334–336) or non-specified ARs prompts endothelial cells to overexpress CD73 (334) as well as the proangiogenic factors VEGF (332, 333, 335, 336), IL-8 (335) and basic fibroblast growth factor (bFGF) (335, 336).

### Tumor Cells

Adenosine binding to ARs on the surface of cancer cells has a profound impact on their biology. For example, the triggering of A1R (337, 338), A2AR (54, 339, 340), and A3R (339, 341–343) induces a variety of cellular responses that augment cancer cell survival such as AKT and ERK1/2 stimulation, as well as Bad inactivation (342). Additional responses to AR signaling contributing to bolstered cancer cell survival include upregulation of Bcl2 (343), downregulation of p53 (338) and Bax (343) as well as aversion of caspase-9 (343) and caspase-3 (54, 343, 344) activation. Paradoxically, extracellular adenosine has also been demonstrated to cause cancer cell death either by setting off A1R (345, 346), A2AR (341, 347), A2BR (348, 349), and A3R (339, 350–354) or *via* induction of AMPK activation upon its cellular uptake and subsequent conversion to AMP (345).

Moreover, A1R (337, 355), A2AR (341, 356), A2BR (344, 357–359), and A3R (343, 360) stimulation augments cancer cell proliferation through activation of PLC (356), protein kinase C-delta (PKC-δ) (356), AKT (356, 357), ERK1/2 (356–360), JNK (356, 358), and p38 (358). Furthermore, triggering of the ARs leads to upregulation of cyclins A (343), B (358), D (343, 358), E (337, 343, 358), estrogen receptor-α (355) as well as downregulation of the cell-cycle inhibitors p27 (337) and p21 (343, 358). Surprisingly, though, activation of A2BR (349) and A3R (341, 350, 353, 361–363) has also been reported to result in a potent cytostatic effect.

Motility (358, 359, 364–369) and invasiveness (358, 359, 367, 370) are additional features of cancer cells that are boosted upon engagement of A1R (364, 365), A2AR (366), A2BR (358, 359, 367, 368), and A3R (369, 370). In terms of mechanisms, signaling initiated by these receptors promotes filopodia formation (367) as well as expression of matrix metalloproteases (MMPs) (358, 359, 370) and FXYD5 (359), a cell membrane glycoprotein known to drive metastasis by reducing cell adhesion (371). In contrast, others claim that A3R triggering hinders the motility and invasiveness of cancer cells (372, 373). Finally, A2AR (374), A2BR (369, 375), and A3R (369, 375–377) stimulation on the surface of cancer cells promotes angiogenesis by boosting secretion of the pro-angiogenic factors VEGF (369, 375, 377), IL-8 (369, 375), angiopoietin 2 (376), and erythropoietin (374).

The contrasting consequences of triggering particular ARs, on the survival, proliferation or migration and invasiveness of tumor cells most probably occur due to the heterogeneity between cells and/or experimental settings employed to assess them. For instance, two different cancer cell lines of distinct tissue origin could have profoundly diverse AR expression profiles as well as different ability to transmit/terminate signaling initiated by these receptors. Moreover, they might have different capacity to produce adenosine, which once released into the medium can trigger ARs in an autocrine fashion. Finally, different concentrations used between experiments, as well as limited specificity of the AR agonists/antagonists, probably constitute additional factors contributing to the observed discrepancies.

## Targeting Adenosinergic Signaling in Cancer Immunotherapy

**Figure 3:**
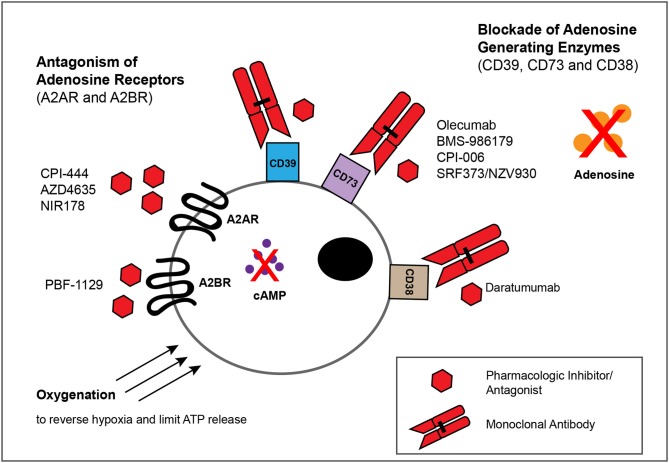
Approaches for blocking adenosinergic signaling in the TME. The inhibitory effects of adenosine in the TME can be circumvented by administration of mAbs or small molecules that target enzymes involved in the catabolism of ATP and NAD, such as CD39,CD73 and CD38, as well as by pharmacologic antagonists of A2AR and A2BR to block adenosine-mediated signaling. Whereas multiple such mAbs and pharmacologic inhibitors/antagonists display antitumor activity within murine models of solid tumors (Tables 1, 2), depicted are only those currently evaluated in patients with solid tumor malignancies (Table 3). Finally, treatments that reduce the extracellular export of ATP, such as oxygenation to reverse hypoxia, can attenuate adenosinergic signaling.

Adenosine confers potent immunosuppressive as well as direct tumor-promoting effects in the TME. Thus, approaches to both blocking its generation and hindering binding to its receptors have become important areas of research (Figure 3). Indeed, extensive pre-clinical experimentation has firmly established that targeting the adenosinergic signaling on its own (Table 1) or in combination with emerging IMTs or established cancer treatments (Table 2) shows important promise and soundly supports the clinical evaluation (Table 3) of these concepts. Here we present an overview of such pre-clinical and clinical studies.

**Table 1:** Evaluation of adenosine-axis blockade in murine models of solid malignancies.

**Target**	**Treatment**	**Tumor model**	**Outcome of adenosine axis blockade depends on presence/unhindered function of**	**Impact on the TME**
CD73	➣ mAbs: TY/23 (368, 378–384) 2C5 IgG2a (384) Oleclumab (385) AD2 (386) ➣ Pharmacologic inhibitor: APCP (378, 379, 381, 383, 384, 387–389)	➣ Breast: 4T1.2 (368, 382, 383) E0771 (368) LM3^a^ (386) MDA-MB-231^a^ (386) ➣ Melanoma: B16-SIY (378) B16-F10 (379, 381, 384, 387, 389) K1735 (381) LWT1 (384) ➣ Ovarian: ID8 (378, 388) ➣ Prostate: TRAMP-C1 (380) RM-1 (382) ➣ Colon: CT26 (385) MC38-OVA (382) ➣ Fibrosarcoma: MCA-induced (380, 382) ➣ Lymphoma: EG7 (379)	➣ Primary tumor expansionrate ↓ (368, 378–382, 385, 387, 389) Host CD73 (387) A2AR on hematopoietic cells (368) T cells, NK cells or B cells (368) T cells (381) CD8 T cells (380) IFN-γ (382) IL-17A (381) Partially retained inmice depleted of B cells (381) Retained in perforin KO mice (382) ➣ Metastasis formation ↓ (368, 379, 380, 384, 386) Retained against tumor cells with significantly reduced CD73 (386) Host CD73 (380) MDSCs (384) Retained in mice depleted of T cells or NK cells (380) Retained in SCID mice lacking T cells, NK cells and functional B cells (386) Host FcRIV (384) FcR binding capacity (384) Independent of the capacity to suppress CD73 catalytic activity (384, 386) ➣ Survival↑ (378, 382, 384, 388)	↑ CD8^+^ T cells (381, 385, 389) ↑ tumor-specific CD8^+^ T cells (382) ↓ CD73 on CD4^+^/CD8^+^ T cells (382, 384) ↑ B cells (381) ↓ Tregs (389) ↑ MDSCs (384) ↓ CD73 on MDSCs (384) ↑ IFN-γ, TNF-α, IL-17A (381) ↓ Ki67^+^ cells (381) ↓ Bcl-2^+^ cells (381) ↓ Microvessel density (383) ↓ VEGF (383)
CD39	➣ mAb: 9-8B (390) ➣ Pharmacologic inhibitor: POM-1 (391)	➣ Melanoma: B16-F10 (391) ➣ Colon: MCA38 (391) ➣ Sarcoma: IGN-SRC- 004^a^ (390)	➣ Primary tumor expansion rate ↓ (391) Host CD39 (391) ➣ Survival ↑ (390) Retained in NOG mice lacking T cells, B cells, NK cells and functional macrophages (390)	
CD38	➣ mAb: NIMR-5 (96) ➣ Pharmacologic inhibitor: Rhein (96)	➣ Lung: 344SQ (96) LLC-JSP (96) 531LN3 (96)	➣ Primary tumor expansion rate ↓ (96) CD8^+^ T cells (96)	↑ CD8^+^ T cells (96) ↑ CD44^hi^CD62L^lo^ CD8^+^ T cells (96) ↓ PD-1^+^TIME3^+^ CD8^+^ T cells (96)↓ Tregs (96) ↓ MDSCs (96)
Intratumoral hypoxia	Respiratory hyperoxia (60% O_2_) (9, 293)	➣ Breast: 4T1 (293) ➣ Melanoma: B16 (9) B16-F10 (293) CL8-1 (9) ➣ Fibrosarcoma: MCA205 (9, 293)	➣ Primary tumor expansion rate ↓ (9) ➣ Metastasis formation ↓ (293) CD4 > CD8 > NK cells (293) Host A2AR (293) Independent of 60%O_2_-induced ROS production (293) ➣ Survival ↑ (9, 293)	↓ Hypoxia (9, 293) ↓ HIF- 1α (9) ↑ FHL-1, FIH-1, VHL (HIF- 1α inhibitors) (9) ↓ CD39, CD73, A2AR, A2BR, COX-2 mRNA (9)↓ extracellular adenosine (9) ↑ CD8^+^, CD69^+^, CD44^+^ cells (293)↓ Tregs (293) ↑ IL-2, IL-12, CXCL9, CXCL10, CXCL11 mRNA (293)↓ TGF-β (293) ↓ FOXP3 in Tregs (293)↓ CD39, CD73, CTLA-4 on Tregs (293) ↑ MHC class I on tumor cells (9) ↓ VEGF, VEGF mRNA (9) ↓ Microvessel density (9)
A2AR	➣ Antagonists: ZM241385 (38, 54) ZM241365 (389) SCH58261 (54, 292, 384, 392–394) FSPTP (395) CPI-444 (396) PBF-509 (397)	➣ Breast: 4T1.2 (292) ➣ Melanoma: B16-F10 (292, 384, 389, 394, 395, 397) CL8-1 (38) BRAF^V600E^-PTEN-deficient mice (393) LWT1 (393) ➣ Colon: CT26 (396) MC38 (396) ➣ NSCLC: PC9^a^ (54) ➣ Bladder: MB49 (395) ➣ HNSCC: Tgfbr1/Pten double KO (392) ➣ Fibrosarcoma: MCA205 (397)	➣ Primary tumor expansion rate ↓ (38, 54, 389, 392, 393, 396) T cells (38) Retained in NUDE micelackingT cells (54) ➣ Metastasis formation ↓ (292, 384, 393, 394, 397) Tumor CD73 (292, 394) Host A2AR (292) T cells, B cells or NK cells (292) Perforin (292) ➣ Survival↑ (384, 396) CD8 T cells > NK cells (394)	↓ CD8^+^, CD4^+^ T cells (395) ↑ CD8^+^ T cells (389, 392, 393) ↑ CD69 on CD8^+^ T cells (393) ↓ A2AR^+^ CD8^+^ T cells (392) ↑ IFN-γ^+^ CD8^+^ T cells (392) ↑ T-bet, 41-BB in/on CD44^+^CD8^+^ T cells (396) ↑ IFN-γ, TNF-α production by CD8^+^ T cells (392) ↑ stimulation-induced IFN-γ/TNF-αproduction by CD8^+^ T cells (396) ↑ NK cells (393, 395) ↑ GzB^+^ NK cells (292) ↓ Tregs (389, 392, 393) ↓ PD-1, LAG3, FOXP3 on Tregs (396) ↓ A2AR^+^ Tregs (392) ↓ FOXP3 (392)
A2BR	➣ Antagonists: PSB1115 (292, 315, 359, 398) ATL-801 (399)	➣ Breast: 4T1.2 (292, 359, 368, 399) E0771 (359) ➣ Melanoma: B16-F10 (292, 315, 359, 398) LWT1 (359) ➣ Bladder: MB49 (399)	➣ Primary tumor expansion rate ↓ (315, 398, 399) Mature T cells (399) T cells (398) Host A2BR (399) Host CXCR3 (399) Retained in A2AR^−/−^ mice (399) Retained in mice depleted of MDSCs but lost upon adoptive transfer of MDSCs (398) ➣ Metastasis formation ↓ (292, 359, 368) Tumor CD73 (292) Retained in RAG^−/−^cγ^−/−^ mice lacking T cells, B cells and NK cells (292) Retained in mice depleted of T cells, NK cells D11c^+^ DCs or macrophages (359) ➣ Survival↑ (359) Tumor A2BR (359) Retained in mice depleted of T cells or NK cells (359)	↑ CD8^+^ T cells (315, 398) ↑ CXCR3^+^ T cells (399) ↑ NKT cells (315, 398) ↓ MDSCs (315, 398)↑IFN-γ, CXCL10 mRNA (399) ↑ IFN-γ, TNF-α, GzB (398) ↓ MCP-1, IL-10 (398) ↓ VEGF (315)↓ Microvessel density (315)

a Patient-derived tumor cell lines, NSCLC, Non-Small-Cell LungCancer. HNSCC, Head and neck squamous cellcarcinoma.

*X > Y: X contributes more than Y to the anti-tumor effect of adenosine axis modulation*.

**Table 2:** Evaluation of concomitant adenosine-axis blockade in murine models of solid malignancies.

**Combinatorial schemes**	**Treatments**	**Tumor model**	**Outcome of concomitant adenosine axis blockade depends on presence/unhindered function of**	**Impact on the TME**
CD73 inhibition & A2AR antagonism	➣ anti-CD73mAb: TY/23 (384) ➣ A2AR pharmacologic antagonist: SCH58261 (384)	➣ Breast: 4T1.2 (384) ➣ Melanoma: B16-F10 (384) LWT1 (384)	➣ Metastasis formation ↓ (384) ➣ Survival ↑ (384) NK cells > CD8^+^ T cells (384) IFN-γ (384) Perforin (partial dependence) (384)	
PD-1 ICB & CD73 inhibition	➣ anti-PD-1mAb: RMP1-14 (382, 385) ➣ anti-CD73mAbs: Oleclumab (385) TY/23 (382)	➣ Breast: 4T1.2 (382) ➣ Colon: CT26 (385) MC38 (382) ➣ Prostate: RM-1 (382)	➣ Primary tumor expansion rate ↓ (382, 385) ➣ Survival ↑ (382, 385)	↑ Tumor-specific CD8^+^ T cells (382, 385) ↑ IFN-γ mRNA (382)
PD-1 ICB & CD38 inhibition	➣ anti-PD-L1mAb: 9G2 (96) ➣ anti-CD38mAb: NIMR-5 (96) ➣ CD38 pharmacologic inhibitor: Rhein (96)	➣ Lung: 344SQ (96) LLC-JSP (96)	➣ Primary tumor expansion rate ↓ (96) ➣ Metastasis formation ↓ (96)	↑ CD8^+^ T cells (96) ↑ CD44^hi^CD62L^lo^ CD8^+^ T cells (96) ↓ PD-1^+^TIME3^+^ CD8^+^ T cells (96) ↑ CD4^+^ICOS^+^ T cells (96) ↓ Tregs (96) ↓ MDSCs (96)
PD-1 ICB & A2AR antagonism	➣ anti-PD-L1: 9G2 (mAb) (96) B7-DC/Fc (400) ➣ anti-PD-1mAb: RMP1-14 (396, 401) ➣ A2AR antagonists: SCH58261 (96, 394, 401) ZM241385 (400) SYN115 (401) CPI-444 (396)	➣ Breast: AT3 (401) 4T1.2 (394, 401) ➣ Melanoma: B16-F10 (394) ➣ Colon: MC38 (396, 401) CT26 (396) ➣ Lung: 344SQ (96) LLC-JSP (96) ➣ Lymphoma: EL4 (400)	➣ Primary tumor expansion rate ↓ (96, 396, 400, 401) IFN-γ (401) Retained in perforin KO mice (401) ➣ Metastasis formation ↓ (394, 401) Tumor CD73 (394) NK cells > CD8^+^ T cells (394) ➣ Survival ↑ (394, 396, 401) CD8^+^ T cells > NK cells (394)	↑ IFN-γ^+^ CD8^+^ or tumor-specific T cells (401) ↑ GzB^+^ CD8^+^ T cells (401) ↑ NK cells (394)
PD-1 ICB & A2BR antagonism	➣ anti-PD-1mAb: RMP1-14 (359) ➣ A2BR antagonist: PSB1115 (359)	➣ Melanoma: B16-F10 (359) ➣ Breast: 4T1.2 (359)	➣ Metastasis formation ↓ (359) ➣ Survival ↑ (359)	
CTLA-4 ICB & CD73 inhibition	➣ anti-CTLA-4mAbs: 9H10 (389) UC10-4F10 (382) ➣ CD73 pharmacologic inhibitor: APCP (389) ➣ anti-CD73mAb: TY/23 (382)	➣ Breast: 4T1.2 (382) ➣ Melanoma: B16F10 (389) ➣ Colon: MC38 (382) ➣ Prostate: RM-1 (382)	➣ Primary tumor expansion rate ↓ (382, 389) CD8^+^ >> CD4^+^ T cells (382) ➣ Survival ↑ (382)	↑ CD8^+^, CD4^+^ T cells (389) ↑ Tumor-specific CD8^+^ T cells (382) ↑ IFN-γ, T-bet mRNA (382) ↑ IFN-γ (389)
CTLA-4 ICB & A2AR antagonism	➣ anti-CTLA-4mAb: 9H10 (389) ➣ A2AR antagonist: ZM241365 (389)	➣ Melanoma: B16F10 (389)	➣ Primary tumor expansion rate ↓ (389)	↑ CD8^+^ T cells (389) ↑ IFN-γ, GzB (389)
CTLA-4 ICB & A2BR antagonism	➣ anti-CTLA-4mAb: UC10-4F10 (359) ➣ A2BR antagonist: PSB1115 (359)	➣ Breast: 4T1.2 (359) ➣ Melanoma: B16-F10 (359)	➣ Metastasis formation ↓ (359) ➣ Survival ↑ (359)	
ACT & CD73 inhibition	➣ T cells: 2C (SIY-specific) (378) Reactive to ID8 (378) OT-I (OVA-specific) (378) ➣ CD73 pharmacologic inhibitor: APCP (378) ➣ anti-CD73mAb: TY/23 (378)	➣ Melanoma: B16-SIY (378) ➣ Ovarian: ID8 (378) ➣ Lymphoma: EG7 (EL4-OVA) (378)	➣ Primary tumor expansion rate ↓ (378) ➣ Survival ↑ (378)	↑ Adoptively transferred T cells (378)
ACT & A2AR antagonism	➣ T cells: anti-HER2 CAR^+^ (135) OT-I (OVA-specific) (388, 396) TDLN-derived (402) Reactive to CMS4 (38) ➣ A2AR antagonists: CPI-444 (396) ZM241385 (38, 135, 402) KW6002 (402) SCH58261 (135, 388)	➣ Breast: E0771- HER2 (135) ➣ Melanoma: B16-OVA (396) ➣ Ovarian: ID8-OVA (388) ➣ Fibrosarcoma: MCA205 (402) 24JK-HER2 (135) ➣ Sarcoma CMS4 (38)	➣ Primary tumor expansion rate ↓ (135, 396) PD-1 ICB (135) IFN-γ (135) ➣ Metastasis formation ↓ (38, 402) Non-myeloablative pretreatment (402) ➣ Survival ↑ (135, 388, 396, 402) PD-1 ICB (135)	↑ Adoptively transferred T cells (396) ↑ Tbet, 41BB, CD69 in/on adoptively transferred CD8^+^ cells (396) ↑ IFN-γ^+^ adoptively transferred T cells (135) ↑ Stimulation-induced IFN-γ, TNF-α production by adoptively transferred CD8^+^ T cells (396) ↑ Stimulation-induced IFN-γ production by adoptively transferred CD8^+^ or CD4^+^ T cells (402) ↑ Tbet, 41BB in/on endogenous CD44^+^ CD8^+^ cells (396) ↑ Stimulation-induced IL-2, IFN-γ, TNF-α production by endogenous CD8^+^CD44^+^ T cells (396)
ACT & intratumoral hypoxia aversion	➣ Respiratory hyperoxia (60%O_2_) ➣ T cells: TDLN-derived (293)	➣ Melanoma: B16-F10 (293) ➣ Fibrosarcoma: MCA205 (293)	➣ Primary tumor expansion rate ↓ (293) Host A2AR (293) ➣ Metastasis formation ↓ (293)	↑ Adoptively transferred T cells (293) ↑ IFN-γ^+^ endogenous/adoptively transferred CD8^+^ T cells (293)
Radiotherapy & CD73 inhibition	➣ Radiotherapy: Single local dose of 20Gy (403, 404) ➣ anti-CD73mAb: Unspecified (403) TY/23 (404)	➣ Breast: TSA (403, 404)	➣ Primary tumor expansion rate ↓ (403, 404) BATF3 (403)	↑ CD103^+^DCs (403) ↑ CD8a^+^ DCs (404) ↑ CD40 on CD8a^+^ DCs (404) ↑ CD8^+^T cells (404) ↑ CD69 on CD8^+^T cells (404) ↑ CD8^+^T cell/Treg ratio (403) ↓ Tregs (404)
Chemotherapy & CD73 inhibition	➣ Chemotherapy: Doxorubicin (405) Paclitaxel (405) ➣ anti-CD73mAb: TY/23(405)	➣ Breast: 4T1.2 (405) AT3 (405)	➣ Primary tumor expansion rate ↓ (405) Partially retained in SCID mice lacking T cells, NK cells and functional B cells (405) CD8^+^ T cells (405) ➣ Survival↑ (405)	↑ Tumor-specific CD8^+^ T cells (405) ↑ IFN-γ (405)
Chemotherapy & CD39 inhibition	➣ Chemotherapy: Mitoxantrone (406) Oxaliplatin (406) ➣ CD39 pharmacologic inhibitor: ARL67156 (406)	➣ Colon: CT26 (406) ➣ Fibrosarcoma: MCA205 (406)	➣ Primary tumor expansion rate ↓ (406) T cells (406) Knockdown of tumor Atg5 (406)	↑ Extracellular ATP (406) ↑ DCs (406) ↑ IFN-γ^+^ CD4^+^, CD8^+^ T cells (406) ↑ IL-17A^+^ γδ T cells (406) ↑ IFN-γ (406)
Chemotherapy & A2R antagonism	➣ Chemotherapy: Doxorubicin (359, 405, 407) Dacarbazine (398) Oxaliplatin (398, 407) ➣ A2R antagonists: SCH58261(A2AR) (405) PSB1115 (A2BR) (359, 398) AB928 (A2AR&A2BR) (407)	➣ Breast: 4T1.2 (405) AT3 (359, 405, 407) ➣ Melanoma: B16-F10 (398)	➣ Primary tumor expansion rate ↓ (398, 405, 407) Tumor CD73 (405) ➣ Survival ↑ (359)	↑ CD8^+^ T cells (398) ↑ Tumor-specific CD8^+^ T cells (407) ↑ NKT cells (398) ↑ GzB (398)
Targeted therapy & CD73 inhibition	➣ anti-ErbB2 mAb 7.16.4 (408) ➣ anti-CD73 mAb TY/23 (408)	➣ Breast: H2N100 (408) TUBO (408) ErbB2-overexpressing mice (408)	➣ Primary tumor expansion rate ↓ (408) Tumor CD73 (408) B cells, T cells or NK cells (408) ➣ Spontaneous tumor formation ↓ (408) ➣ Metastasis formation ↓ (408) ➣ Survival ↑ (408)	↑ CD8^+^ T cells (408) ↑ CD4^+^ FOXP3^−^ T cells (408) ↓ MDSCs (408)
Targeted therapy & A2AR antagonism	➣ BRAF inhibitor: PLX4720 (393) ➣ MEK inhibitor: Trametinib (393) ➣ A2AR antagonist: SCH58261 (393)	➣ Melanoma: BRAF^V600E^-PTEN-deficient mice (393) BRAF^V600E^ LWT1 (393)	➣ Primary tumor expansion rate ↓ (393) ➣ Metastasis formation ↓ (393)	

*TDLN, tumor-draining lymphnode*.

**Table 3:** Clinical evaluation of adenosine-axis targeting in patients with solid tumors.

**Molecular target**	**Clinical Trial identifier**	**Agents**	**Phase**	Design overview^*^	**Solid tumor indications**	**Sponsor**	**Launched on**
CD73	NCT02503774	Oleclumab	I	➣ Single agent	Advanced solid malignancies	MedImmune	2015
				➣ In combination with durvalumab (anti-PD-L1)			
	NCT03736473	Oleclumab	I	➣ Single agent	Advanced solid malignancies	AstraZeneca	2018
	NCT03773666	Oleclumab	I	➣ In combination with durvalumab (anti-PD-L1)	Muscle-invasive Bladder Cancer	Dana-Farber Cancer Institute	2018
	NCT03267589	Oleclumab	II	➣ In combination with durvalumab (anti-PD-L1)	Relapsed ovarian cancer	Nordic Society for Gynecologic Oncology	2018
	NCT03334617	Oleclumab	II	➣ In combination with durvalumab (anti-PD-L1)	PD-1/PD-L1 inhibition-resistant NSCLC	AstraZeneca	2018
	NCT03742102	Oleclumab	Ib/II	➣ In combination with durvalumab (anti-PD-L1) and paclitaxel (chemotherapy)	Metastatic Triple Negative Breast Cancer	AstraZeneca	2018
	NCT03611556	Oleclumab	Ib/II	➣ In combination with gemcitabine (chemotherapy) and nab-paclitaxel (chemotherapy)	Metastatic pancreatic cancer	MedImmune	2018
				➣ In combination with gemcitabine and nab-paclitaxel and durvalumab (anti-PD-L1)			
				➣ In combination with mFOLFOX (chemotherapy regimen comprising oxaliplatin, leucovorin, 5-FU)			
	NCT03381274	Oleclumab	Ib/II	➣ In combination with osimertinib (EGFR^T790M^inhibitor)	Advanced NSCLC	MedImmune	2018
				➣ In combination with AZD4635 (A2Aantagonist)			
	NCT02754141	BMS-986179	I/IIa	➣ Single agent	Advanced solid malignancies	Bristol-Myers Squibb	2016
				➣ In combination with nivolumab (anti-PD-1)			
				➣ In combination with rHuPH20 (drug deliveryenzyme)			
	NCT03454451	CPI-006	I/Ib	➣ Single agent	Advanced solid malignancies	Corvus Pharmaceuticals	2018
				➣ In combination with CPI-444 (A2Aantagonist)			
				➣ In combination with pembrolizumab (anti-PD-1)			
	NCT03549000	NZV930	I/Ib	➣ Single agent	Advanced solid malignancies	Novartis	2018
				➣ In combination with spartalizumab (anti-PD-1)			
				➣ In combination with NIR178 (A2Aantagonist)			
				➣ In combination with NIR178 andspartalizumab			
CD38	NCT03473730	Daratumumab	I	➣ Single agent	Metastatic Renal Cell Carcinoma or Muscle Invasive Bladder Cancer	M.D. Anderson Cancer Center	2017
A2A	NCT02403193	NIR178	I/Ib	➣ Single agent	Advanced NSCLC	Palobiofarma	2015
				➣ In combination with spartalizumab (anti-PD-1)			
	NCT03207867	NIR178	II	➣ Single agent	Advanced solid malignancies	Novartis	2017
				➣ In combination with spartalizumab (anti-PD-1)			
	NCT03742349	NIR178	Ib	➣ In combination with spartalizumab (anti-PD-1) and LAG525(anti-LAG3)	Triple-negative Breast Cancer	Novartis	2018
	NCT02655822	CPI-444	I/Ib	➣ Single agent	Advanced solid malignancies	Corvus Pharmaceuticals	2016
				➣ In combination with atezolizumab (anti-PD-L1)			
	NCT03337698	CPI-444	Ib/II	➣ Single agent	Metastatic NSCLC	Hoffmann-La Roche	2017
				➣ In combination with atezolizumab (anti-PD-L1)			
	NCT02740985	AZD4635	I	➣ Single agent	Advanced solid malignancies	AstraZeneca	2016
				➣ In combination with durvalumab (anti-PD-L1)			
A2B	NCT03274479	PBF-1129	I	➣ Single agent	Advanced NSCLC	Palobiofarma	2018

NSCLC, non-small-cell lung cancer.

* *Mentioned are schemes comprising at least one adenosine-axis modulator*.

### Blockade of Adenosine Generation

As previously described, CD73 is an nucleotidase that converts AMP, generated from CD39- or CD38/CD203-mediated catabolism of ATP or NAD respectively, to adenosine. Its central role in adenosine generation is underscored by the fact that CD73-deficient mice display drastically decreased interstitial levels of adenosine, not only at steady state, but also upon induction of trauma or hypoxia (409, 410). CD73 knock-out mice exhibit hindered tumor growth and metastatic spreading (378–380, 387) and mice inoculated with tumor cells lacking CD73 survive longer than mice inoculated with tumor cells expressing this ecto-enzyme (378, 388). Indeed, administration of anti-CD73 monoclonal antibodies (mAb) (368, 378–386) or of a CD73-specific pharmacologic inhibitor (378, 379, 381, 383, 384, 387–389) impairs tumor growth (368, 378–382, 385, 387, 389) and metastasis (368, 379, 380, 384, 386) while increasing survival (378, 382, 384, 388). Of note, CD73 can also act as an adhesion/signaling molecule to promote metastasis in a catalytic-activity independent manner (386, 411, 412). Mechanistically, the aforementioned treatments have been shown to promote intra-tumoral accumulation of CD8^+^ T cells (381, 382, 385, 389), B cells (381) as well as of Th1- and Th17-associated cytokines (381) while decreasing the levels of intra-tumoral VEGF (383) and the presence of Tregs (389). Of note, even though metastasis can be modestly inhibited by anti-CD73 therapy in an immune-system independent fashion (368, 386), most of the antitumor effect of CD73 blockade is due to alleviation of A2AR-mediated immunosuppression (368).

No doubt encouraged by these pre-clinical studies, four anti-CD73 mAbs are currently being evaluated as monotherapies in small scale trials targeting a variety of solid tumors. In July 2015, MedImmune launched a first in-human trial (NCT02503774) evaluating the human anti-CD73 mAb Oleclumab, which allosterically prevents CD73 from assuming its catalytically active conformation (413). In June 2016, Bristol-Myers Squibb (BMS) launched a Phase I/IIa trial (NCT02754141) to assess the efficacy of BMS-986179, a human IgG2-IgG1 hybrid mAb that not only inhibits CD73-exerted AMP hydrolysis but also induces CD73 internalization (414). In April 2018, Corvus Pharmaceuticals initiated clinical evaluation (NCT03454451) of their humanized anti-CD73 mAb, CPI-006, which directly competes with AMP for the CD73 active site (415). Finally, in July 2018, Novartis listed a Phase I/Ib trial (NCT03549000) evaluating the efficacy of SRF373/NZV930, a human mAb that impedes CD73 activity *via* a currently undisclosed mechanism, and was pre-clinically developed by Surface Oncology before being exclusively licensed to Novartis for further clinical development.

CD39 also critically contributes to the generation of extracellular adenosine from ATP as evidenced by the fact that deficiency of this enzyme results in significantly decreased adenosine content in tissues, not only at steady state, but also upon ischemia induction (80). Similar to studies with CD73-deficient mice, tumor growth and metastasis are reduced in CD39-null mice (391, 416). In addition, intraperitoneal delivery of a CD39 inhibitor in immunocompetent mice reduces tumor growth rates (391). Administration of an anti-CD39 mAb increased the survival of immuno-deficient mice inoculated with patient-derived tumors (390), indicating that CD39 can also promote tumor growth or metastasis in an immune system independent manner. In terms of mechanisms, several studies have demonstrated that *in vitro* inhibition of CD39 activity by pharmacologic inhibitors (45, 47, 62) or blocking mAbs (45, 417, 418) results in enhanced functionality of T cells (45, 47, 62, 418) and NK cells (45, 47, 418), as well as decreased Treg-mediated suppression of T cell proliferation (47, 417). Even though restriction of CD39 activity *in vitro* conclusively alleviates adenosine-induced immunosuppression, a surprisingly small number of studies demonstrate effectiveness of this approach within tumor-bearing mice. Finally, while humanized mAbs targeting CD39, such as IPH52 (Innate Pharma) have been developed, clinical studies exploring CD39 blockade/inhibition have not been launched.

As previously mentioned, the concerted activity of CD38 and CD203a, can functionally replace CD39 toward the generation of extracellular adenosine. Further substantiating the soundness of CD38-blockade as a cancer treatment, immunocompetent CD38-null mice display reduced tumor growth (419) whereas tumors devoid of this ectonucleotidase grow slower both in immuno-competent (96) as well as in immuno-deficient mice (97). Indeed, administration of CD38 mAbs retards tumor growth (96, 420). Interestingly, tumors derived from anti-CD38 mAb-treated mice encompass more CD8^+^ T cells and less Tregs and MDSCs (96). Moreover, increased fraction of CD8^+^ T cells infiltrating these tumors display an effector memory phenotype while less of these cells are double positive for the exhaustion markers PD-1 and TIM3 (96). Three anti-CD38 mAbs, Daratumumab (Janssen Biotech), Isatuximab (Sanofi), and MOR202 (Morphosys) are being clinically evaluated. Daratumumab was FDA-approved in 2015 for treating multiple myeloma patients, while to date the most advanced testing of Isatuximab and MOR202 as monotherapies are respectively the Phase II trials NCT01084252, NCT02960555, and NCT02812706, as well as the Phase I/IIa trial NCT01421186. Of note, in addition to modulating the enzymatic activity of CD38, these mAbs also have the capacity to induce cytotoxicity through diverse mechanisms, such as induction of complement activation, Ab-dependent cellular cytotoxicity (ADCC) or phagocytosis, and programmed cell death (420). Albeit extensive clinical experience of utilizing the aforementioned mAbs against CD38-overexpressing hematologic malignancies, the recently launched trial NCT03473730 constitutes the first application of a CD38-specific mAb in patients with solid tumor malignancies.

Another approach for limiting the intratumoral interstitial adenosine is the oxygenation of the TME (293). As mentioned, hypoxia promotes build-up of extracellular adenosine at least by inducing upregulation of CD39 and CD73 as well as downregulation of adenosine transporters. Indeed, in pre-clinical models, respiratory hyperoxia (60% oxygen) lowers intra-tumoral adenosine levels (9), tumor growth rates (9), metastasis formation (293) and increases survival of tumor-bearing mice (9, 293). Mechanistically, this treatment boosts MHC-I levels on the tumor-cell surface (9), the presence of CD8^+^, CD69^+^, or CD44^+^ cells within the TME (293) and reduces the presence of Tregs (293) as well as the latter's capacity to express CD39, CD73, CTLA-4, or FoxP3 (293). Moreover, increased oxygenation of tumors not only averts angiogenesis through reduction of VEGF concentration (9), but also dampens expression of molecules associated with immune dysfunction, such as TGF-β, CD39, CD73, A2AR, A2BR and COX-2 (9, 293), the rate-limiting enzyme of PGE_2_ biosynthesis, while increasing the mRNA levels of pro-inflammatory agents, such as IL-2, and IL-12a (293).

### Blockade of Adenosine Receptor Binding

Along with blocking adenosine production with small molecules or mAbs, another approach to inhibit adenosine-induced signaling is to directly block binding to its receptors A2AR and A2BR. Underscoring the potent protumoral effect of A2AR-trigerring, mice devoid of this receptor present reduced rates of tumor growth and metastasis, and in some instances tumors undergo complete rejection (38, 292, 400, 402). In addition, administration of pharmacologic A2AR antagonists recapitulates the anti-tumor effects of A2AR-deletion since it results to reduced primary tumor expansion (38, 54, 389, 392, 393, 396) and metastasis formation (292, 384, 393, 394, 397) ultimately leading to prolonged survival (384, 396). Mechanistically, tumors derived from A2AR-antagonist-treated mice are more heavily infiltrated by CD8^+^ T cells (389, 392, 393) as well as NK cells (389, 392, 393) and encompass fewer Tregs (389, 392, 393). In addition, *in vivo* A2AR antagonism leads to increased expression of CD69 (393), T-bet (396), and 4-1BB (396) as well as production of IFNγ and TNFα (392, 396) by intra-tumoral CD8^+^ T cells. Furthermore, this intervention increases the fraction of intra-tumoral NK cells producing GzB (292) and reduces the expression of PD-1, LAG3, FoxP3 and A2AR by tumor-infiltrating Tregs (392, 396). Interestingly, the A2AR antagonists ZM241385 and SCH58261 exhibit the capacity to curb primary tumor growth even in a T cell-independent manner (54). Notably, A2A antagonism *in vivo* increases activation induced cell death (AICD) of intra-tumoral T cells (395), a finding corroborating observations that cAMP-accumulation in the T cell cytosol averts terminal effector differentiation and AICD (421, 422). Three A2AR antagonists are currently being evaluated as single agents in Phase I/II trials to treat cancer patients bearing solid tumors. In particular, Corvus Pharmaceuticals, AstraZeneca, and Novartis have undertaken the clinical development of CPI-444 (NCT02655822), AZD4635 (NCT02740985), and NIR178 (NCT02403193, NCT03207867), respectively.

As for A2AR, genetic deletion of A2BR reduces tumor growth rate (399, 423) while A2BR^−/−^ tumor cells display reduced metastatic potential (359, 367). Notably, administration of A2BR antagonists in tumor-bearing mice reduces tumor growth (315, 398, 399) and metastasis (292, 359, 368) eventually prolonging their survival (359). Mechanistically, antagonism of A2BR *in vivo* augments the intra-tumoral presence of CD8^+^ T cells (315, 398), NKT (315, 398) as well as the mRNA levels of IFNγ and CXCL10 (399) and the concentration of TNFα, IFNγ, and GzB (398) in the TME. This intervention further results in decreased accumulation of MDSCc (315, 398) and IL-10 (398), as well as reduced levels of VEGF and angiogenesis (315). Based on encouraging preclinical results, Palobiofarma recently launched a dose escalation Phase I study (NCT03274479) administering PBF-1129, a selective A2BR inhibitor, in patients with advanced Non-Small Cell Lung Cancer (NSCLC).

### Combinatorial Treatment Approaches

Since multiple ecto-enzymes with redundant functions contribute toward extracellular adenosine production and both A2AR and A2BR triggering mediate the majority of adenosine's pro-tumoral effects, monotherapies may not be sufficient to block the adenosine-signaling axis. In addition, there is strong rationale for combination with IMTs, such as ICB of PD-1/PDL-1 or CTLA-4, as well as ACT, radiotherapy and chemotherapy, to further unleash the cytotoxic capacity of T cells, which, as will be discussed, can become highly sensitized to adenosine-mediated immunosuppression.

#### Combinations of Adenosine-Axis Blockade Agents

Concurrent mAb-mediated (418) or pharmacologic (47) inhibition of CD39 and CD73 failed to potentiate CD73-blockade-induced suppression of adenosine production by Tregs and ovarian cancer cell lines. These findings are corroborated by the observation that skin biopsies derived from CD39^−/−^CD73^−/−^ mice have identical capacity to produce adenosine upon injury induction with counterpart biopsies derived from CD73^−/−^ mice (424).

Alone the same lines, others addressed whether simultaneous blockade of CD73 and of A2AR would result in higher anti-tumor efficacy. Of note, CD73^−/−^A2AR^−/−^ mice present superior tumor control as compared to single knockout mice (384). Moreover, tumors in A2AR-null mice express twice as much CD73 at their core when compared to tumors formed in wild-type mice (384). Indeed, dual therapy with an anti-CD73 mAb and an A2AR agonist confers superior tumor protection as compared to either one as a monotherapy (384). However, this additive effect is lost when CD73 is targeted with a pharmacologic inhibitor, thus underscoring the capacity of CD73 to promote tumor progression in a catalytic activity-independent manner (384). In light of these studies, Evotec and Exscientia have partnered to develop A2AR/CD73 bi-specific inhibitory molecules (425), whereas NCT03454451, NCT03549000 as well as the Phase Ib/II clinical trial NCT03381274 sponsored by MedImmune all include solid tumor-bearing patient cohorts scheduled to be treated with combinations of an anti-CD73 mAb along with a pharmacologic A2AR antagonist.

#### Adenosine-Axis and PD-1 Blockade

Briefly, PD-1 is an immunosuppressive receptor that upon binding to its ligands, PDL-1 and PDL-2, dampens T-cell activity thereby enabling tumors to evade immune-destruction. Blockade of the PD-1-PDL-1/2 signaling axis results in durable complete responses in the clinic for a fraction of treated patients (1), and many pre-clinical and clinical studies have explored concomitant inhibition of adenosine production, or antagonism of A2AR and A2BR, to improve response rates.

It has been demonstrated that CD73^+^ tumor cells are resistant to PD-1 ICB (401) and that simultaneous mAb-mediated blockade of CD73 and PD-1 synergistically enhances tumor control and survival in mice (382, 385). Mechanistically, the dual therapy augments intra-tumoral CD8^+^ tumor-specific T cells (382, 385) and IFNγ mRNA levels (382) as compared to single-agent treatments. Several clinical trials assessing anti-CD73 mAb treatment along with anti-PD-1 mAb (NCT03454451, NCT03549000) or anti-PDL-1 mAb (NCT02503774, NCT03773666, NCT03267589, NCT03334617) of advanced solid tumors are recruiting or underway. Intra-tumoral upregulation of CD38 and subsequent adenosine production was recently identified as a mechanism of acquired resistance to PD-1/PD-L1 blockade and mAb-mediated or pharmacologic inhibition of CD38 was shown to significantly improve the anti-tumor efficacy of an anti-PDL-1 mAb (96). In terms of mechanisms, tumors from mice receiving the combinatorial therapy displayed higher accumulation of CD8^+^ T cells, effector memory CD8^+^ T cells, ICOS^+^ CD4^+^ T cells and lower levels of MDSCs and Tregs as compared to tumors from single-agent treated mice (96).

The potential for synergy between the co-administration of A2R antagonists with anti-PD-1 mAb is underscored by the observations that PD-1 blockade enhances A2AR expression on tumor-infiltrating CD8^+^ T cells (401), as well as that PD-1 blockade is more efficacious, in terms of increasing the survival of tumor-bearing mice, when these mice lack the A2AR (400). Vice versa, A2AR triggering on the surface of CD8^+^ T cells derived from tumor tissue (382), tumor draining lymph nodes or spleen (396) promotes PD-1 expression suggesting that simultaneous PD-1 blockade would boost the anti-tumor efficacy of A2A antagonism. Indeed, several groups demonstrated that concurrent provision of PD-1 checkpoint inhibitors along with A2AR antagonists is more effective than single-agent treatments at reducing tumor growth rate (96, 396, 400, 401) and metastasis formation (394, 401), as well as at improving survival (394, 396, 401). Moreover, the combination enables increased production of IFNγ and GzB by CD8^+^ tumor infiltrating T cells (401) while augmenting the intra-tumoral presence of NK cells (394). Five clinical trials for the treatment of solid-tumor patient cohorts with A2AR antagonists along with anti-PD-1 Ab (NCT02403193, NCT03207867) or anti-PD-L1 Ab (NCT02655822, NCT03337698, NCT02740985) are ongoing. Finally, dual therapy comprising A2BR antagonism and PD-1 blockade is superior to either monotherapy at decreasing metastasis and improving survival of tumor-bearing mice (359). However, no clinical trials have been launched to date to explore this combination in human cancer patients.

#### Adenosine-Axis and CLTA-4 Blockade

The blockade of CTLA-4, an immune checkpoint receptor predominantly expressed by T cells and which competes with the co-stimulatory receptor CD28 for binding to CD80/CD86 on the surface of antigen presenting cells (APCs), has also generated durable clinical responses in advanced cancer patients (1). Tumor-bearing mice receiving CTLA-4 blockade and pharmacologic (389) or Ab-mediated (382) inhibition of CD73 display superior tumor control (382, 389) and overall survival (382) than counterparts receiving single agent treatments. Mechanistically, these dual therapies are more effective than corresponding monotherapies at increasing the intra-tumoral presence of tumor-specific CD8^+^ T cells (382), CD4^+^FoxP3^neg^ T cells (389) as well as the levels of IFNγ (389) and of mRNA coding for IFNγ and T-bet (382). Likewise, concomitant provision of CTLA-4 ICB and antagonists of either A2AR (389) or A2BR (359) leads to decreased tumor growth (389) and metastasis formation (359), as well as to higher survival of tumor-bearing mice (359) when compared to single treatments. In terms of mechanisms, combining CTLA-4 ICB with an A2AR antagonist augments intratumoral CD8^+^ T cell presence as well as IFNγ and GzmB levels (389).

#### Adenosine-Axis Blockade and Adoptive T Cell Therapy

There are two main approaches to ACT. Either autologous tumor-reactive T cells are expanded from tumor biopsies prior to patient re-infusion [i.e., tumor infiltrating lymphocyte (TIL) therapy], or peripheral blood T cells are gene-engineered to express a tumor-specific T cell receptor (TCR), or a so-called chimeric antigen receptor (CAR; a fusion protein that links scFv-mediated tumor antigen-binding with intracellular endo-domains associated with T cell activation). Cancer patients are typically lymphodepleted prior to ACT, and following infusion they receive high doses of IL-2, both of which support T cell engraftment (426). TIL therapy has achieved robust and durable responses in advanced melanoma patients, while CAR therapy targeting CD19 has yielded unprecedented clinical responses against a variety of advanced, treatment-refractory B cell malignancies (118, 427, 428).

Synergy has been demonstrated between strategies limiting adenosine production blockade and ACT within tumor-bearing mice. Indeed, ACT confers increased control of tumors lacking CD73 expression (388) and dual therapy of ACT and pharmacologic or mAb-mediated inhibition of CD73 was more robust than single treatments at augmenting tumor control and overall survival (378). Mechanistically, pharmacologic inhibition of CD73 potentiated the anti-tumor efficacy of ACT at least by boosting the homing of the adoptively transferred tumor-specific T cells at the tumor sites (378). Likewise, respiratory hyperoxia in mice increased the ability of adoptively transferred T cells to curb primary tumor expansion and metastasis formation by augmenting their capacity to accumulate in the TME and produce IFNγ (293).

Similarly, A2AR deficiency (402) or siRNA-mediated suppression of A2AR and A2BR expression (38) on the surface of adoptively transferred T cells leads to enhanced prevention of metastatic spreading (38, 402) and improved survival of tumor-bearing mice (38). Several groups have validated these observations by demonstrating that ACT and concomitant administration of A2AR antagonists is superior to single treatments in terms of decreasing tumor growth (135, 396), hindering metastasis formation (38, 402) and ultimately improving survival (135, 388, 396, 402). Interestingly, others claim that A2AR antagonism improves the efficacy of adoptively transferred CAR^+^ T cells only if PD1 ICB is co-administered (135). In terms of mechanisms, concomitant A2AR antagonism not only increases intra-tumoral presence of adoptively transferred T cells (396) but also elevates their activation status. In particular, when A2AR antagonists were co-administered, tumor-derived, adoptively transferred or endogenous CD44^+^ CD8^+^ T cells, exhibit heightened expression levels of T-bet, 4-1BB, and CD69 (396) while demonstrating increased capacity to produce IFNγ and TNFα (135, 396, 402).

#### Adenosine-Axis Blockade Combined With Radiotherapy, Chemotherapy or Targeted Therapies

It is well documented that radiotherapy (RT) as well as several chemotherapeutic (CT) drugs have the capacity to induce ATP release (406, 429–433). Since such regimens also elevate the expression levels of CD39 (405, 407, 434) and CD73 (405, 407, 435–437), it is possible that the concentration of interstitial adenosine in the TME rises sharply upon application of these treatments. Therefore, several investigators have explored whether concomitant provision of agents targeting the adenosine axis increase the anti-tumor efficacy of RT or of various CT agents.

Indeed, mAb-mediated inhibition of CD73 increased the anti-tumor efficacy of RT (403, 404) and this synergistic effect was even more apparent upon concurrent CTLA-4-blockade (404). Mechanistically, CD73 inhibition increases the presence of CD8^+^ T cells as well as of CD8α^+^ or CD103^+^ DCs within irradiated tumors while decreasing Tregs (403, 404). Moreover, concomitant CD73 blockade was shown to increase the activation status of CD8^+^ T cells and CD8α^+^ DCs within irradiated tumors as evidenced by the elevated expression levels of CD69 and CD40, respectively (404). Likewise, concurrent mAb-mediated inhibition of CD73 (405) or pharmacologic blockade of CD39 activity (406) boosted the tumor control (405, 406) and survival (405) of mice treated with the CT drugs Doxorubicin (405), Paclitaxel (405), and Mitoxantrone (406). Of note, such dual therapies were shown to not only augment intra-tumoral presence of DCs (406) and tumor-specific CD8^+^ T cells (405) but also the fraction of intra-tumoral CD4^+^ or CD8^+^ T cells producing IFNγ (406) as well as the levels of IFNγ in the TME (405, 406). In light of such observations, the clinical trials NCT03611556 and NCT03742102 are set to decipher the potency of CT regimens when provided in combination with the CD73-blocking Ab Oleclumab, supplemented or not by PD-1 blockade.

Along the same lines, others explored if direct antagonism of A2AR and A2BR would augment the antitumor effects of CT agents. Indeed, tumor-bearing mice treated with Doxorubicin (359, 405, 407), Dacarbazine (398), or Oxaliplatin (398, 407) in combination with A2AR (405), A2BR (359, 398), or dual A2AR/A2BR antagonists (407) displayed superior tumor control (398, 405, 407) or survived longer (359). Of note, tumors derived from mice treated with the combination of Dacarbazine and PSB1115, an A2BR antagonist, were more heavily infiltrated by CD8^+^ T cells as well as NKT cells and contained higher levels of GzB than tumors derived from counterpart mice subjected to Dacarbazine monotherapy (398). Likewise, concomitant administration of AB928, a dual A2AR and A2BR antagonist, along with Doxorubicin or Oxaliplatin increased the intra-tumoral detection of tumor-specific CD8^+^ T cells (407).

Finally, others have sought to decipher whether adenosine axis blockade enhances the anti-tumor efficacy of particular targeted therapies. For instance, it has been recently demonstrated that high expression levels of CD73 in tumors derived from breast cancer patients are associated with resistance to Trastuzumab, an anti-HER2/ErbB2 mAb, and that artificial CD73 overexpression promotes resistance to Trastuzumab-like therapy in immunocompetent murine models of breast cancer (408). Subsequently, the authors moved on to show that when such mice receive dual therapy comprising anti-CD73 and anti-ERB2 mAbs they exhibit inferior tumor expansion rate as well as reduced metastatic spreading and survive longer than counterpart mice treated with either single agent treatments (408). In terms of mechanisms, the combinatorial therapy significantly increases the intra-tumoral presence of CD8^+^ and CD4^+^FoxP3^neg^ T cells while decreasing MDSCs (408). In addition, melanoma patients harboring BRAF-mutant tumors exhibit a trend for elevated expression of CD73 whereas co-administration of an A2AR antagonist in mice bearing BRAF-mutant tumors increased the therapeutic benefit achieved either by BRAF inhibition or by the combination of BRAF and MEK inhibitors (393). Finally, CD73 and A2AR are overexpressed in NSCLCs harboring EGFR mutations (438) and even though preclinical studies demonstrating increased efficacy of concomitant inhibition of EGFR and A2AR are not currently publicly available, the clinical trial NCT03381274 includes a cohort of patients with advanced NSCLC that will receive both an EGFR inhibitor and an A2AR antagonist.

## Summary and Future Perspectives

Adenosine is critically involved in a range of physiologic processes including wound healing, and its levels are tightly regulated under homeostatic conditions. In solid tumors, however, adenosine concentration is significantly elevated, predominantly due to stress-induced ATP release coupled with the overexpression of nucleotidases, such as CD39 and CD73 that contribute to its catabolism. Primarily by engaging A2AR and A2BR, also overexpressed in the TME as a result of hypoxia and inflammation, adenosine diminishes the activity of protective immune infiltrates, such as T cells, NK cells and DCs, while boosting the inhibitory capacity of immunosuppressive subsets, including Tregs and MDSCs. For instance, A2AR and A2BR-induced cAMP accumulation within T cells blunts their differentiation, proliferation, cytokine production and target cell killing, predominantly through PKA activation. Along with establishing an anti-inflammatory and tolerogenic TME, adenosine also promotes blood vessel formation and assists tumors in subverting adjacent fibroblasts to further support tumor growth and metastasis.

Administration of small molecules or mAbs with the aim to block adenosine-signaling, either by limiting its production or its binding to ARs, has yielded important tumor control in various pre-clinical tumor models. Moreover, simultaneous blockade of adenosine production and receptor binding, achieved by an anti-CD73 mAb co-administered with an A2AR antagonist, for example, have demonstrated it synergy. Given the potent suppression of T cells by adenosine, it comes as no surprise that increases in tumor control and survival conferred by ICB (anti-PD-1 and anti-CTLA-4 mAbs) or ACT, is significantly enhanced by concomitant administration of agents countering the adenosine axis. Synergy of such adenosine axis modulators has further been shown with RT, as well as CTs, schemes known to promote immunogenic cell death (i.e., ATP is released), as well as with some targeted therapies.

While blockade of adenosine production and A2AR/A2BR antagonism are being tested in the clinic as monotherapies, increasing numbers of clinical trials combining adenosine-signaling blockade with IMTs or classic treatment approaches (i.e., RT, CT and targeted therapies) are recruiting and/or underway. Given the important responses achieved by a proportion of patients to immunotherapeutic-regimens, and the tremendous levels of immunosuppression mediated by adenosine, the development of existing or new agents targeting this axis, along with further testing of combinatorial strategies, is warranted. Indeed, targeting the adenosine axis holds great promise in the improved treatment of cancer patients.

## Author Contributions

GC, MI, DA, and SV conceived the manuscript. DA, SV, and MI drafted the manuscript. GC, PR, CM-C, and CC reviewed the manuscript and provided feedback, and MI revised the manuscript. MI, DA, and SV made the figures, and DA assembled the tables.

### Conflict of Interest Statement

The authors declare that the research was conducted in the absence of any commercial or financial relationships that could be construed as a potential conflict of interest.
